# Perceptions and Attitudes of Gynecologic and Pediatric Professionals Regarding Dietary Exposure to Chemical Pollutants

**DOI:** 10.3390/ijerph17113946

**Published:** 2020-06-02

**Authors:** Juan Pedro Arrebola, Araceli Muñoz, Silvia Ferrero, Cristina Larrea-Killinger

**Affiliations:** 1 Department of Preventive Medicine and Public Health, University of Granada, 18071 Granada, Spain; 2 Instituto de Investigación Biosanitaria de Granada (ibs.GRANADA), 18012 Granada, Spain; 3 CIBER de Epidemiología y Salud Pública (CIBERESP), 28029 Madrid, Spain; larrea@ub.edu; 4 School of Social Work, University of Barcelona, 08035 Barcelona, Spain; aracelimunoz67@gmail.com; 5 Food Observatory, Department of Social Anthropology, University of Barcelona, 08001 Barcelona, Spain; 6 Obstetrics and Gynecology, Sant Joan de Déu University Hospital, 08950 Barcelona, Spain; sferrero@sjdhospitalbarcelona.org

**Keywords:** risk perception, attitudes, health professionals, chemical pollutants, food, mercury, bisphenol A, pesticides

## Abstract

There is increasing concern regarding the potential implications of continuous dietary exposure to low doses of artificial chemical pollutants, particularly in critical life stages such as pregnancy and lactation. Within a wider social research, we analyzed the risk perception, discourses, and attitudes of health professionals regarding dietary exposure to artificial chemical contaminants. Data was collected by personal interviews on 35 health professionals from two Spanish regions. Although the participants’ discourses were strongly dominated by the nutritional composition and microbiological contamination, 34 expressed some concern regarding metals, and 23 regarding pesticides. Although only one participant mentioned a plasticizer (i.e., bisphenol A), we noted an underlying concern, since six professionals admitted to recommending pregnant women to somewhat avoid plastic food containers, and were aware of mother-to-child transmission and accumulation of artificial chemicals. The ubiquity of the exposure, the inability to locate the threat, and contradictory messages can all create a sense of helplessness and subsequent cognitive adjustments. Our participants also reported a lack of information, particularly on emerging pollutants. In conclusion, we found a range of valuable discourses that can aid in orienting public health strategies aimed at health professionals who have a substantial influence on their patients.

## 1. Introduction

Widespread human exposure to environmental chemicals has been well-documented in a number of bio-monitoring studies [1,2]. These chemicals include substances with very diverse chemical structures and uses, from those that are highly persistent and bioaccumulative in the food chain and within organisms, to others that are readily metabolized and excreted from the human body [3,4]. There are diverse pathways through which a contaminant can enter the body, but the most significant source of a large number of them is through food [5].

During the last few decades, there has been increasing concern and uncertainty about the potentially harmful effects of continuous exposure to low doses of these contaminants, even when well below the toxicity thresholds established by regulatory agencies. This has posed a challenge for traditional toxicological models, which are based on the study of high levels of exposure [6]. There is currently a considerably uncertainty of the health effects at current exposure levels, mainly because of the timing between exposure and the effect, as well as the potential synergies between exposure to different contaminants with similar mechanisms of action [7,8]. This problem, in combination with several probabilistic issues in regards to the development of pathologies significantly influences the population’s perceptions about the associated risks and contributes to making it “psychologically distant” [9].

The general population receives periodic information campaigns based on the results of research. However, conflicting information from different sources can induce emotional stress, resulting in uncertainty or even distrust [10]. These tensions are very important since they can change individuals’ perception of risks and their resulting attitudes [11]. This is particularly the case for those groups that are highly vulnerable to exposure [12] such as pregnant and breastfeeding women [13,14], who already may have fears of the existing threats to the health of their children from various external factors [15]. These groups are also subject to the traditional gendered nature of the family, social pressure on “good mothering” [16], and a “medicalization process” of pregnancy [17], as well as the social amplification of risk [18]. In this regard, the role of health professionals is crucial for the proper development of pregnancy and during maternal breastfeeding [19], as they represent a very important reference for women during these periods of time. In fact, it has been suggested that information campaigns aimed at professionals could be even more effective than those focused on pregnant and breastfeeding women since, in addition to disseminating information, health professionals could contribute to the evaluation of the possible consequences of exposure and to a more precise estimation of risks, among other areas [20]. These professionals are also key elements for the transference of scientific knowledge to the general population. Despite the abovementioned, health professionals do not usually receive specific training in this area [20].

Even though scientific literature has been traditionally dominated by quantitative studies, qualitative approaches can provide unique information that cannot be achieved by using other approaches [21,22]. Indeed, when following established standards for scientific rigor, qualitative research is ideal for exploring social events as experienced by individuals in their natural context [23], becoming essential for addressing specific aspects of health research [24].

This paper aims to analyze the risk perception, discourses, and attitudes of health professionals regarding exposure to chemical contaminants, with emphasis on the food pathway.

## 2. Materials and Methods

This study is part of a wider project (CSO2014-58144-P) focused on the analysis of the discourses and practices related to food consumption and the presence of chemical substances in food. This qualitative approach has been acknowledged as valid for understanding underlying behaviors, attitudes, and perceptions related to health [25]. The target population was pregnant and breastfeeding women, as well as health professionals involved in these women’s care. The project explored the social and cultural basis of trust and distrust among these women and how responsibility for the possible effects of these substances on health is assigned.

**Figure 1:**
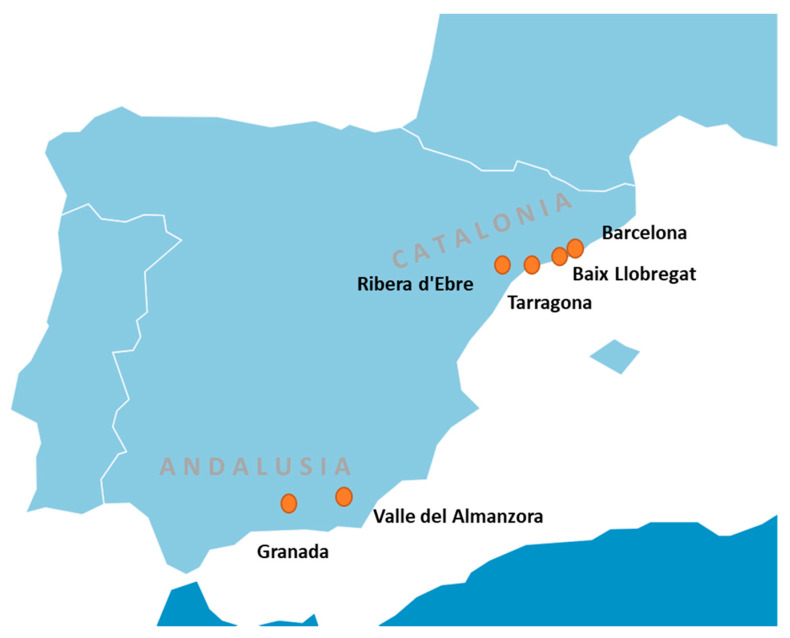
Recruitment locations of the study population.

The present research includes a qualitative study based on semi-structured interviews with health professionals actively involved in gynecology and pediatric departments. Semi-structured interviews consist of a series of specific questions in which participants have substantial flexibility for answering. This allow participants to express themselves in their own way and using their own words [26]. Thus, it is a type of flexible interview where the order and details of how the questions are asked can be varied, allowing the participant to develop their ideas and speak widely on the issues raised [27]. The study was performed in six different locations from two Spanish autonomous regions: Catalonia (Barcelona and metropolitan area, Baix Llobregat, Tarragona, and Ribera d’Ebre) and Andalusia (Granada and surroundings, Valle del Almanzora-Almería) (Figure 1). Since the two regions have marked sociodemographic, lifestyle, health, and economic differences [28,29], this approach was intended to capture heterogeneity in the discourses as well as to assess potential regional differences. Access to these health professionals was possible through five public hospitals and four primary health care centers. The objective was to compare their discourses regarding their perceptions about chemical risks in food consumption.

The interview process began in January 2016, and the last interviews were completed in September of 2017. All the participants were informed about the research objectives and methods, and written informed consent was obtained from each of them.

Sample selection was intentional and purposive and was based on the specific parameters of the study following the sample saturation criterion [30] and a strategy of maximum variation. The aim of this approach was to obtain the largest representativeness and to capture the existing discursive diversity [31]. The semi-structured interview script is provided as supplementary material.

In total, we conducted 35 interviews with health professionals, exploring, among other issues, the dangers and trust associated with foods containing added chemicals. In addition, the interviews addressed the possibility of bioaccumulation and/or mother-to-child transmission of these substances. We also inquired about their knowledge of chemical contaminants and the possibility of avoiding or reducing exposure in everyday life as well as the dietary advice they usually give to pregnant and breastfeeding women. Lastly, we also asked the interviewees if they thought that health professionals should receive more information regarding the artificial chemical substances present in food in order to offer better information to pregnant and breastfeeding mothers, and to establish what would be the most important areas to address. We recruited a total of 18 medical doctors, 7 nursing professionals, 5 midwives, and 5 professionals with both nursing and midwife degrees (Table 1).

This research was conducted in accordance with the Declaration of Helsinki, and the protocol was approved by the ethic committees of each of the participant institutions (2015/6459; Conresmum; PIC-16-16; P15-135; 2015/6459/l). All the participants were informed about the study characteristics and signed an informed consent.

## 3. Results

### 3.1. Chemical Substances Present in Food and Their Transmission

The most frequently mentioned contaminants were metals and, in particular, mercury, which many participants associated with the consumption of fish. In total, 34 participants expressed concern about metal exposure.

*“Well, we’ve received information now about tuna; we shouldn’t eat too much canned tuna, which has a high concentration of mercury, of heavy metals.”*

(Midwife)

Another frequently mentioned group of contaminants were pesticides, to which 23 participants expressed concern. However, no participant showed concern to any specific pesticide subtype. During the interviews, bisphenol A was only mentioned by one professional, and no one expressed concerns on phthalates or other plasticizers. However, a total of six participants acknowledged their worry about the potential health implications of plastic food containers.

For the study participants, there are substances that the body cannot eliminate or can do so only partially, since they bioaccumulate and may affect the health of the pregnant woman, the fetus (particularly during the first trimester of pregnancy), and the breastfeeding infant. Some health professionals showed certain knowledge on the potential health effects and the mechanisms of action of contaminants (e.g., the central nervous system and hormonal effects). They overall think that any chemical contaminant can pass into the placenta and into breast milk, just as with alcohol or medication. Therefore, they think of pregnancy and breastfeeding as stages in which there is a transfer of these chemicals from the mother to the fetus/infant.

*“It’s like with medications; there are medications that cross the placental barrier, or the lactation barrier, and others that don’t. But certainly, there will be many that do. Of course, a lot of them will.”*

(Nurse)

*“I think that these substances accumulate, in fat tissue especially, and they’re eliminated in breastfeeding.”*

(Doctor)

All the participants said that they were aware of the apparent universal nature of human exposure to contaminants. For them, most foods have some artificial chemical component, used mainly to improve preservation, the organoleptic characteristics, or to increase production. These compounds are mainly present in processed foods, including industrial baked goods, soft drinks, and farmed fish.

*“Sausage also, to preserve it, and to give it a certain taste, also contain them. Food coloring in yogurt, also, for example. What would be odd is to say what they don’t contain”*

(Doctor)

*“Well, all industrial baked goods, for sure. And, well, pretty much everything that needs to last contains preservatives (…) some chemical part. That is, except for fruit and, in principle, the cuts of meat you buy; most things already have them, of the food you buy, all of it has some kind of chemical component to preserve it”*

(Doctor)

### 3.2. Health Risks Related to Exposure through Diet

Regarding their perceptions of the link between diet and health, the participants’ arguments are strongly influenced by the nutritional properties of foods, by the potential biological risk for the pregnant or breastfeeding woman, and, to a lesser extent, by the presence of artificial chemicals. As a result, their discourses about healthy eating focus predominantly on purely nutritional issues such as sugar, fat and alcohol content, and the presence of microorganisms and/or parasites (e.g., anisakis), as well as allergies and intolerances (e.g., gluten, lactose). Thus, for example, they discourage the consumption of fatty and raw products, as well as those containing sauces.

*“Well, I stress to them, regarding fish, freezing, anisakis”*

(Doctor)

*“Meat, because the fats we get with meat, because they are saturated fats and aren’t good either”*

(Midwife)

In general, our health professionals tended to trust on fruit, vegetables, grains, pulses and pasta, as they are “healthier and free of toxins.”

*“Trust? I don’t know. Well, fruit and vegetables, and everything that contains protein, things that aren’t very fatty and so on, because I think they’re always better, right? Or that they’re not, I mean, prefabricated food. Everything you buy in a supermarket that’s already prepared in factories, well, all of that I don’t think is safe, of course.”*

(Nurse)

*“Well, grains, also, are a food that I trust. They’re good for you, rich in vitamins, fiber. For the pregnant woman, it’s a very complete food, and I believe not enough importance is given to grains in this country. Grains and also pulses. They seem to me to be two good foods for pregnant women and for the population in general.”*

(Nurse)

In addition, study participants also expressed a trust in milk and other dairy products, especially those that are fat-free or have a low fat content, but only when they are properly packaged. In this respect, food packaging was considered to improve the perception of product quality and thus minimizes the risk of health damage.

*“Dairy products may be among those in which I trust the most. Yes, dairy products, I think they’re good food. They’re packaged well, well pasteurized, I believe they’re beneficial; they are good for you. I see dairy as a healthy food for everyone. Especially if it’s low-fat or fat-free.”*

(Midwife)

Fish, meat, as well as processed and fast foods, on the other hand, were the most frequently mistrusted food items. Professionals very often associated fish consumption with exposure to mercury, and meat consumption with the hormones and steroids used to accelerate animal growth. Regarding chicken, we evidenced two contradictory messages since it is perceived as a “healthy food” because of its low fat content but, on the other hand, it was frequently associated with dangerous artificial hormones.

*“Because I have the feeling, or it’s my understanding, that in some places they use certain hormones to increase the animals’ weight; this remains in the meat. And of course, that passes into the mother’s milk and then to the child.”*

(Physician)

It is interesting that packaging, which in dairy products or fruit is associated with an element of protection, was also associated with processed foods and increased exposure to substances that are dangerous or harmful to health.

*“I mean, the less prepared, the less packaged, uh, the more I can see it, the better.”*

The majority of participants admitted to being aware of chemical exposure through diet and were wary of pre-cooked foods, or foods that are prepared/produced by unknown sources, because “they contain the highest amount of artificial chemicals”. However, they considered the dose as a determining factor in terms of harmful effects, since most of them expressed that eating very little of these foods or not eating them very often would prevent the harmful effects, or that these effects would be so subtle that they would be outweighed by the benefits of the food item.

*“Maybe for me, it’s not a lack of trust, but the fact that someone else is cooking for me, that I don’t know how it was cooked, or where it came from, well, every once in a while, it’s no big deal.”*

(Midwife)

*“Well, more than distrust, I just don’t think it’s good to eat them on a regular basis. For example, prepared, prefabricated, precooked meals […] nothing happens if you do it every once in a while.”*

(Nurse)

*“Especially food that is packaged and industrialized; that’s what I say no to. From time to time if they have a craving, they can do it, but they’re not a good habit for a pregnant woman on a daily basis.”*

(Midwife)

It is probably because of the idea that there are “safer” exposure doses that some professionals consider a varied diet to be the best way of reducing the harmful effects of chemical exposure. In fact, some of them showed a distrust on the potential health effects in the general population at current exposure levels. One person even thought that perhaps small, frequent doses of certain toxins could help a person’s immune system, as with vaccines.

*“Well, I think it’s very difficult to purify everything and […] And sometimes, purifying is perhaps worse than eating a little poison from time to time to immunize yourself and to build defenses.”*

(Midwife)

The most frequently mentioned chemical substances in food items, and of greatest concern to health professionals, were preservatives, metals, pesticides, and synthetic hormones. As mentioned earlier, among chemical contaminants, there is a clear concern about mercury and other metals in fish.

These health professionals are aware that exposure to human-made chemicals through food could have immediate effects, such as birth defects (e.g., some are aware of the history of thalidomide), but they were also concerned about the long-term effects (e.g., hormonal imbalances, fertility problems, allergies, and cancer), as the fetus and baby are perceived to be highly vulnerable.

*“Yes, there is a difference between what they can cause. Occasional acute food poisoning can lead to gastroenteritis, an acute gastrointestinal condition, while the accumulation of these substances over the long term can lead to hormonal imbalances, endocrine imbalances, which can lead to more serious illnesses.”*

(Doctor)

One participant also mentioned a proper environmental pathology, i.e., Multiple Chemical Sensitivity. On the other hand, participants also acknowledged that there is great uncertainty about the possible effects of exposure to chemicals during pregnancy or breastfeeding.

*“And that gives me the feeling that they have a lot of chemicals, you know? A lot of products that maybe we don’t know much about what they are, or what consequences they will have in the long run. In the short run, for sure, but in the long run, we don’t know where all this will lead.”*

(Doctor)

Probably as a strategy to minimize the problem, some health professionals argued that we have been exposed to chemicals throughout our entire lives.

*“All our lives we’ve drunk, I mean, there wasn’t any bottled water, but the plumbing was lead, man, lead, you know? And the thing is, that’s how it was, you know, I remember playing with mercury from the thermometers when they broke, until the ball broke completely, which was a celebration when it broke. Well, that’s it, I don’t know. My father smoked in the car all his life, the car smelled like smoke. I mean, I’ve smoked more in his car than in clubs. Well, that’s the way it is, that’s the way it is.”*

(Doctor)

In this respect, they tended to relate longevity with health, which they use to argue that the problem of chemicals in food may not be so relevant.

*“Hey, other people have eaten preservatives their whole lives, and my grandmothers are 97 years old.”*

(Midwife)

However, older people tended to point to the distant past (when they were children, or when their parents were children) as a better time, when everything was more natural, and there was a lower exposure to chemicals.

*“So I think we’re eating all that a lot, and we don’t know how much it affects everyone’s health. I mean, I realize that we’re not eating the same natural foods we ate fifty years ago. It’s all been manipulated.”*

(Midwife)

*“Now we have a lot more variety, but maybe having more variety […], isn’t any better either, is it? We have more access to more food, but that doesn’t mean we eat better.”*

(Doctor)

One participant, in fact, points to the exposure as the price we have to pay to live in today’s society.

*“This is 2015; right now we’re in Barcelona, and it’s true, I believe we have to pay a price for being able to eat good food, lots of food, and of relatively good quality, and practically any time of the year.”*

(Doctor)

Finally, and despite the fact that most of the participants admitted to being aware of the potential threats of the exposure to public health, many of them perceived this as a secondary issue in comparison to other more pressing and well-documented problems that should be more urgently targeted.

*“We have other known problems, and we don’t solve them. I mean, it’s all very well, yeah, all right. But many people are dying of malaria, we know what they’re dying of, friends, and we don’t do what we need to do, millions of people die every year. So, well, this thing with lead and so on, it’s bad, but there are other issues that are more at the forefront.”*

(Doctor)

### 3.3. Trust in Production Processes and Authorities

The vast majority of professionals largely trusted the regulatory and legislative processes of the Spanish health authorities, and believed that they would never allow a product to be marketed if it was not completely safe.

*“Pregnant women are advised to avoid anything that […], especially dairy products, or animal products, that can be bought directly from the producer. That is, they shouldn’t drink raw milk or cheese that hasn’t been pasteurized […], sausages straight from the butcher, without having undergone […], inspections […]”*

(Doctor)

*“Health controls […] I hope so because otherwise, you don’t have peace of mind. You trust, especially, in a place with all the guarantees, it doesn’t matter if it’s a large or small shop, it needs to be a place with all the guarantees. For me, that means you are selling what you can sell, that it’s been verified, it’s been analyzed, it’s safe food. That’s why you don’t trust someone who’s selling food on the street, without any kind of permit. Because it hasn’t been monitored by anyone.”*

(Doctor)

It has, indeed, been suggested that improving public health inspections could have negative consequences, as this will have an impact on the price of products and would therefore make them less accessible.

*“How much are you willing to pay for products to be better? This is easy. We’ll triple the audits, investigations, and we’ll get rid of even more when it comes to quality standards. I’ll tell you what’s going to happen. This is going to mean more expensive products. Are you willing for that to happen? I don’t know if I am. That’s the way things are.”*

(Doctor)

We also evidenced a feeling of helplessness in the face of the pervasiveness of exposure, and a tendency to avoid considering the seriousness of the problem by pointing out that untrusting quality controls would make their life too hard.

*“To me, […] well, it seems sad, you know? That we have to have this, but it seems like that is where society is headed, I mean, I don’t really know if it can be avoided, you know?”*

(Midwife)

Interestingly, the perception of the quality of controls is very high among persons whose friends or relatives run food-related businesses subject to health controls, but not as high for the only person who previously worked as a health inspector.

*“There’s a lot of criticism of kebab meat [….] But then I heard that they have to be supervised by health authorities, too.”*

(Midwife)

*“I suppose that the health inspector who goes and checks it, well he will do a good job, right, just like I try to do my job as well as I can, and I know, well I imagine it’s true that the institutions responsible for this go through the factories, or through the places where food is prepared and they will inspect what they have to, and they’ll do it well. It’s the same, for example, with dairy products, I have family who has a farm, and I know for sure that, for example, the quality controls on milk are very strict.”*

(Doctor)

Only two participants expressed no trust for the authorities and the quality control system in general, citing as examples palm oil and petroleum derivatives that have not been withdrawn from the market even though they are harmful.

*“For example, palm oil is in a whole lot of foods. If it’s carcinogenic, they should take it out. Why do they keep on putting palm oil in food, if it is carcinogenic?”*

(Nurse)

With regard to labelling, we found a general trust about the information on food packaging labels, particularly those that claim they do not contain added chemicals.

*“When they say the food is free of substances, you feel a sense of confidence. Which is also transmitted to the general population.”*

(Doctor)

Some participants, however, doubted about the truthfulness of “BIO” or “Organic” labels and even argued that the industry “takes chemicals off the label” to make more money. They also indicated that organic products are of better quality and have better organoleptic characteristics, but that if non-organic products are allowed by the authorities, it is because they are not harmful. Despite this, they expressed some distrust toward foods grown in greenhouses, and this was especially the case among the participants from the south-eastern region (the largest area devoted to intensive agriculture in the country). Interestingly, and despite the abovementioned disparities between the study regions, this was the only appreciable inter-regional difference in the discourses of the participants.

*“But really, I come from a place that is full of greenhouses and pesticides. I know that the vegetables are also contaminated […] I don’t think we eat anything that’s healthy!”*

(Midwife)

Eating home-grown food, or knowing the person in charge of the garden where your vegetables come from, leads to much more trust in the foods consumed, as does proximity to their place of origin. This is particularly the case when the product was grown in Spain, since participants believed that there are better controls in Spain than in Latin America, Asia, or Africa.

*“Well, if you buy in a butcher’s shop that you trust and you buy liver, and you know it’s been raised in the mountains, eating grass, you have a lot more guarantee.”*

(Doctor)

*“No, because I don’t know if Asian restaurants pass all the safety inspections or if the products that come through….from ships, in Asian restaurants that have warehouses in Madrid, I don’t know if they have, if they pass the health inspections [….] they must pass.”*

(Midwife)

The participating health professionals expressed a substantial confidence in scientific studies and, in fact, called for more research to study the effect of exposure to contaminants, and for research findings to be published in general media. They also called for more uniformity in the information, as they complained about receiving contradictory messages, especially through the Internet. They found the information published in the written press to be more truthful. One participant expressed doubts about the correct design and interpretation of the results of scientific studies.

*“Well, it’s hard to isolate yourself from this, first of all. I would like to be better informed about the studies that prove what is said about this, right?, because [….] well, maybe because of my professional training, I like to first do a critical reading, right, of what’s published. Not everything that is published is true, reliable, or verifiable. I mean, I would like to know the source and how these conclusions have been reached, in order to know to what extent I can believe what’s being said.”*

(Nurse)

### 3.4. Avoiding Exposure

In general, there was a sense of vulnerability regarding the health threats from chemicals in the environment, since they are believed to be present in most products of everyday use and can affect everyone, making it impossible to avoid them. One of the participants stated that it is up to the consumer to decide what to buy and thus, we can influence industrial production. The majority of the participants considered the authorities as having ultimate responsibility for protecting the general population. They believed that any substance that generates any kind of danger should be prohibited, with no exception.

On the other hand, many emphasized the enormous lack of knowledge related to the subject and called for more training on the matter. Information should be very categorical, focused on knowing “which food products are good and which are not”.

*“What I don’t know is how we can know if they are good or not”*

(Doctor)

The participants showed a strong association between healthy/good quality foods and high prices. In general, they found it very difficult to have a toxic-free diet since it would involve eating non-processed foods, which are more expensive and do not last as long. In addition, they found the information on food labels to be extremely hard to understand. They also pointed out that, in Spain, organic food is very expensive for the average consumer, and they perceived that this is not the case in other European countries.

Lastly, they also expressed particular doubts surrounding the recommendations that they should make to pregnant and breastfeeding women, as they are wary of the real magnitude of the problem of exposure and think that these women already have too much information about healthy living habits, and that this causes psychological stress.

*“It is horrible that they cannot eat anything (…) they have anxiety (…) I think in the end they are going to have such a poor and restricted diet”*

(Doctor)

## 4. Discussion

Although, in our study population, the perception of our participating healthcare professionals regarding healthy foods was strongly dominated by nutritional composition and microbiological contamination, a substantial number of participants acknowledged being concerned about artificial chemical pollutants. Indeed, the most frequently mentioned contaminants were metals and, specifically, mercury, which the majority of the participants associated with fish consumption. This perception is in accordance with the recommendations of the European Food Safety Authority (EFSA), the Directorate-General for Heath and Food Safety (DGSANCO) of the European Commission, and the Spanish Agency for Consumer Affairs, Food Safety and Nutrition [32]. In addition, a considerable number of participants expressed their concerns regarding dietary pesticide exposure. Interestingly, although certain plasticizers have gained attention over the last years, only one health professional mentioned any of these chemicals (specifically bisphenol A). However, we noted an underlying unspecific concern regarding these chemicals, since six professionals acknowledged being aware of the potential dangers of plastic exposure and declared suggesting to their patients to avoid in some way plastics in food containers. Interestingly, these concerns had implications in their daily clinical practice.

The participants had strongly internalized the concepts of mother-to-child transmission and accumulation of chemical substances, acknowledging a potential health risk in some cases, and citing as examples widely-known cases in medicine related to teratogenic drugs such as thalidomide [33]. There is a bodily representation of toxicity and exposure, perceiving the body as a reservoir of toxic chemical substances [34]. However, reaction to the exposure to contaminants in foods was different to the reaction towards drugs for various reasons. On the one hand, the ubiquity of the exposure, the inability to locate the threat, and the contradictory messages received can all create a sense of helplessness, as natural processes such as eating or breathing are perceived as risky activities [34]. For example, oily fish contains polyunsaturated fatty acids, which can be beneficial to neuronal development, but they also accumulate a large quantity of mercury, which could negatively impact the neurodevelopment of the baby. This often leads to the development of cognitive adjustments to minimize feelings of stress, including skepticism towards the messages, distancing oneself from the problem, or relegating all responsibility to controls [16].

These observations are consistent with those from a previous study by our research group in the city of Barcelona, where we found that our sample population perceived public institutions to have two main responsibilities: 1) to raise awareness of the risks from chemical substances, and 2) to control and monitor the exposure. While institutions may meet these responsibilities, responsibility also remains in the hands of individuals [34].

On the other hand, it is possible to identify in the discourses both “good actors” (health authorities, scientists), that look after the safety of the consumer, as well as “bad actors” (the industry), that try to deceive in order to obtain higher profits. Another observed strategy for dealing with exposure to chemicals is the attribution of, or belief in, a certain immunological effect from very limited exposure, as found by Crighton and colleagues among pregnant women [16]. Additionally, the fact that there is a considerable gap between exposure and effect, and that we are referring to pathologies with multiple potential causes, contributes to reducing the sense of danger or to prioritizing other more immediate problems.

On the other hand, our study reveals that professionals are aware of the information overload that women receive during pregnancy and lactation, as well as the responsibility women feel they have, which is related to the gender role that society assigns them [35]. Therefore, many professionals advocate adapting specific messages to different population groups and weighing whether the benefits of information really compensate for the stress faced by mothers in the face of said information, especially considering the lack of adequate resources to protect themselves [36]. This raises the need for providing clear and concise information, which includes practical solutions to each problem, and not to explain risks if these cannot be avoided.

Additionally, the interviewed health professionals claim for specific categorical information from the scientists and authorities, including which foods are “good” and which are “bad” for human health. Currently, the perception of risk in the population is highly influenced by the increasing levels of industrialization in the food chain, which has led to the existence of an enormous distance between food production and consumption, with the consequent risk of the presence of artificial chemical substances in any step in the chain [37]. Therefore, in recent decades there has been a clear shift from a focus on the quantity of food and the avoidance of short term risk, towards a concern for healthy food and possible long term risks [38]. In fact, previous literature have acknowledged a current shift from a focus on wealth in “a society of scarcity” towards a focus on “the distribution of risk” in modern society [39]. This risk is not limited to a specific place, and therefore, can be universal, impossible to perceive by our senses, and very hard to measure [40]. This phenomenon has led to a change in the relationship between the population and health/scientific authorities, from whom more research and information is needed regarding the effects of exposure to contaminants [38]. However, it is interesting to observe that some people distrust the methodological quality of scientific research and information, which is consistent with Beck’s argument that, in the risk society, there is suspicion that information is omitted by regulatory and scientific authorities which therefore leads individuals to become “small, private alternative experts” [41]. The magnitude of this issue is amplified in the case of health professionals, and in particular, among those with certain research experience, which provides them with a certain confidence in distrusting methodological aspects of research studies. This process also results on the creation of a “forced” or “conditional” trust toward public experts [41]. In this context, natural chemistry is perceived as something positive for human health and the environment, and artificial chemistry as something potentially harmful to the organism, even if there is a lack of mechanistical understanding [34,42,43].

## 5. Conclusions

Considering the limited research in this field to date, our study offers relevant data concerning perspectives and attitudes of Spanish health professionals towards the issue of dietary exposure to chemical pollutants. We found a range of valuable discourses that can aid in orienting public health strategies aimed at health professionals, who have substantial influence on their patients. Particularly, their discourses evidenced a lack of information and awareness of emerging pollutants such as bisphenol A, that should be considered for further public health campaigns. In light of these results, we would also like to stress the need for multidisciplinary teams when it comes to dealing with the problem of human exposure to environmental contaminants, which should integrate both specialists in basic sciences and epidemiology, as well as experts in the social sciences.

## Figures and Tables

**Table 1:** Description of the study participants.

Occupation	Place of Residence	Sex
Nurse	Catalonia	Female
Doctor	Catalonia	Male
Doctor	Catalonia	Female
Nurse	Catalonia	Female
Nurse	Catalonia	Female
Midwife	Catalonia	Female
Nurse	Catalonia	Female
Nurse	Catalonia	Female
Midwife	Catalonia	Female
Doctor	Catalonia	Female
Doctor	Catalonia	Male
Nurse	Catalonia	Female
Doctor	Catalonia	Male
Doctor	Catalonia	Female
Doctor	Catalonia	Male
Doctor	Catalonia	Female
Doctor	Catalonia	Male
Doctor	Catalonia	Female
Doctor	Catalonia	Male
Nurse midwife	Catalonia	Female
Nurse midwife	Catalonia	Female
Doctor	Catalonia	Female
Doctor	Catalonia	Female
Nurse midwife	Catalonia	Female
Nurse midwife	Catalonia	Female
Doctor	Catalonia	Female
Midwife	Catalonia	Female
Midwife	Catalonia	Female
Nurse	Catalonia	Female
Nurse midwife	Andalusia	Female
Doctor	Andalusia	Male
Doctor	Andalusia	Female
Doctor	Andalusia	Female
Midwife	Andalusia	Female
Doctor	Andalusia	Female
